# Transcatheter self-expandable heart valve implantation for aortic stenosis with coexisting Kommerell diverticulum with right-sided aortic arch

**DOI:** 10.1093/ehjcr/ytae314

**Published:** 2024-07-02

**Authors:** Sho Takagi, Masanori Yamamoto, Masafumi Saito, Yoshihiro Goto

**Affiliations:** Department of Cardiovascular Surgery, Toyohashi Heart Center, Toyohashi, Aichi, Japan; Department of Cardiology, Toyohashi Heart Center, Toyohashi, Aichi, Japan; Department of Cardiology, Nagoya Heart Center, Nagoya, Aichi, Japan; Department of Cardiology, Gifu Heart Center, Nishigifu, Gifu, Japan; Department of Cardiology, Toyohashi Heart Center, Toyohashi, Aichi, Japan; Department of Cardiovascular Surgery, Toyohashi Heart Center, Toyohashi, Aichi, Japan

An 87-year-old woman with severe aortic stenosis (AS) was referred to our hospital. Due to her age and comorbidities, transcatheter aortic valve replacement (TAVR) was planned. However, baseline computed tomography angiography (CTA) revealed a Kommerell diverticulum (KD) with a right-sided aortic arch (RAA) and tortuosity of the descending aorta extending to the abdominal aorta (*Figure 1A–C*). Additionally, the aortic valve CTA showed significant calcium proliferation from the annulus to the left ventricular outflow tract (*Figure 1D–F*). Echocardiography confirmed a narrowed aortic valve opening with a mean gradient of 67 mmHg (*Figure 1G*). Based on the CTA findings, a 26 mm Evolut-FX (Medtronic) self-expanding transcatheter heart valve (SE-THV) was chosen. The SE-THV was delivered via the femoral artery using a Confida stiff guidewire (Medtronic) and a 14 French-compatible InLine sheath. This sheath passed through the abdominal aorta, descending RAA, and KD (*Figure 1H–J*) without encountering any resistance. The SE-THV was implanted without vascular injury (*Figure 1K*), and the intraoperative mean gradient across the valve was significantly decreased (*Figure 1L*). The post-operative echocardiogram showed a significant reduction in the mean gradient to 6 mmHg (*Figure 1M*). Aortic stenosis and concomitant KD with RAA are a rare developmental abnormality of the aorta.^1^ To our knowledge, this is the first reported case of severe AS successfully treated with TAVR in a patient with KD and RAA. The Evolut-FX’s single-spine design, intended to improve deliverability, may have contributed to the procedural success. Despite the challenges associated with transfemoral TAVR, the latest generation SE-THV enabled a smooth procedure.

**Figure 1:**
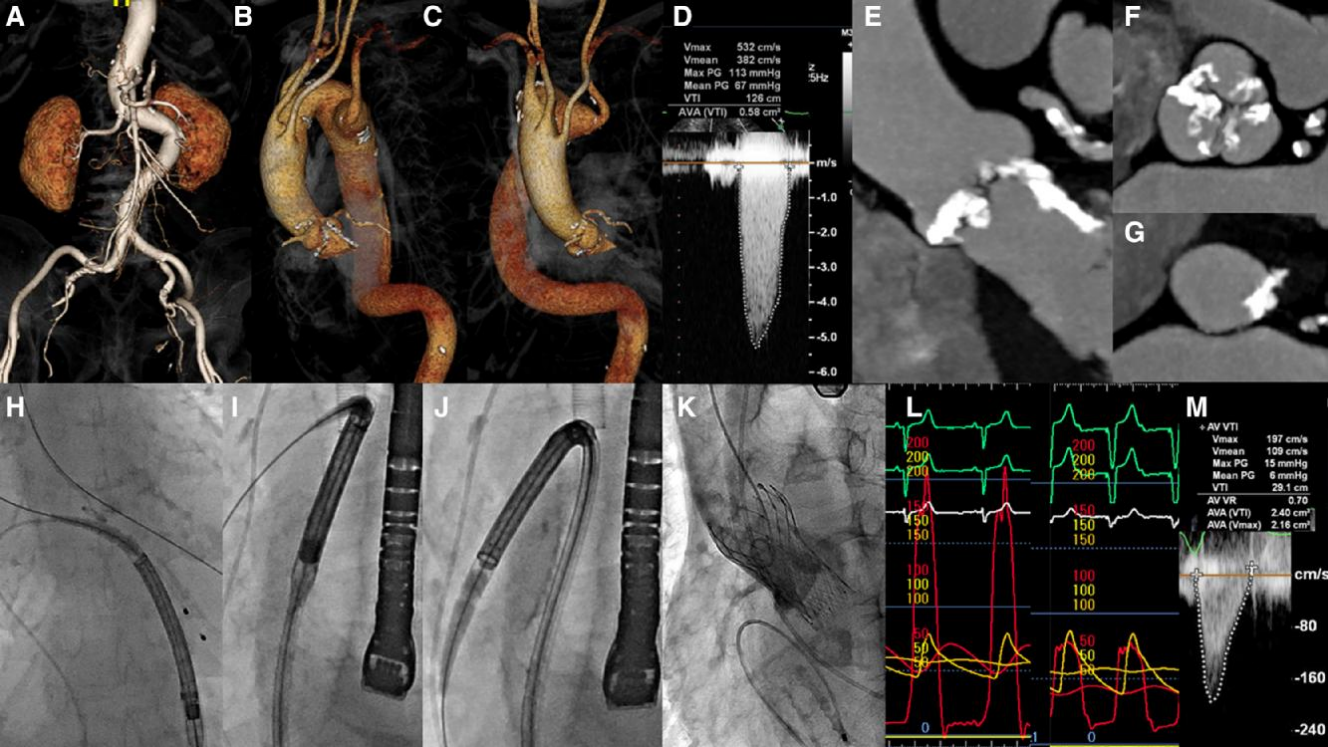
Tortuosity of abdominal aorta (*A*), aberrant left subclavian artery and right subclavian artery. Kommerell diverticulum with a right-sided aortic arch (*B*), a strong angulation and from the descending to the abdominal aorta was sharply curved (*C*), echocardiographic finding confirmed severe aortic stenosis (*D*), baseline computed tomography angiography demonstrated an excessive calcium proliferation was evident from the annulus to the left ventricle outflow excessive calcification (*E* and *F*), annular area of 357 mm^2^, an annular perimeter of 66.7 mm (*G*), the 14 French-compatible InLine sheath successfully passed through the abdominal aorta with the support of a Confida stiff guidewire (*H*), the Evolut-FX successfully transversed the sharply curved descending aorta and the severely angulated Kommerell diverticulum with a right-sided aortic arch (*I* and *J*), the Evolut-FX was successfully implanted (*K*), resulting in an improved post-operative haemodynamics (*L*) and echocardiogram showing a mean gradient of 6 mmHg (*M*).

**Consent:** Written informed consent was obtained from the patient for publication of this case report and accompanying images.

**Funding**: None declared.

## Data Availability

The data underlying this article are available in the article.
